# Cancer Disparities Among Alaska Native People, 1970–2011

**DOI:** 10.5888/pcd11.130369

**Published:** 2014-12-18

**Authors:** Janet J. Kelly, Anne P. Lanier, Teresa Schade, Jennifer Brantley, B. Michael Starkey

**Affiliations:** Author Affiliations: Anne P. Lanier, Teresa Schade, Jennifer Brantley; B. Michael Starkey, Alaska Native Tribal Health Consortium, Alaska Native Epidemiology Center, Division of Community Health Services, Anchorage, Alaska.

## Abstract

**Introduction:**

Cancer is the leading cause of death among Alaska Native people. The objective of this study was to examine cancer incidence data for 2007–2011, age-specific rates for a 15-year period, incidence trends for 1970–2011, and mortality trends for 1990–2011.

**Methods:**

US data were from the Surveillance, Epidemiology, and End Results (SEER) Program SEER*Stat database and from the SEER Alaska Native Tumor Registry. Age-adjusted cancer incidence rates among Alaska Native people and US whites were compared using rate ratios. Trend analyses were performed using the Joinpoint Regression Program. Mortality data were from National Center for Health Statistics.

**Results:**

During 2007–2011 the cancer incidence rate among Alaska Native women was 16% higher than the rate among US white women and was similar among Alaska Native men and US white men. Incidence rates among Alaska Native people exceeded rates among US whites for nasopharyngeal, stomach, colorectal, lung, and kidney cancer. A downward trend in colorectal cancer incidence among Alaska Native people occurred from 1999 to 2011. Significant declines in rates were not observed for other frequently diagnosed cancers or for all sites combined. Cancer mortality rates among Alaska Native people during 2 periods, 1990–2000 and 2001–2011, did not decline. Cancer mortality rates among Alaska Native people exceeded rates among US whites for all cancers combined; for cancers of the lung, stomach, pancreas, kidney, and cervix; and for colorectal cancer.

**Conclusion:**

Increases in colorectal screening among Alaska Native people may be responsible for current declines in colorectal cancer incidence; however; improvements in treatment of colon and rectal cancers may also be contributing factors.

## Introduction

Cancer is the leading cause of death among Alaska Native (AN) people (1). Before the mid-1900s, cancer was considered a rare disease among AN people, but since then, cancer incidence has increased dramatically (2). Reports of salivary and esophageal cancers in the 1960s showed emerging patterns of cancer among Eskimo people in Alaska (3–7). During that time, cancer mortality overall did not differ from mortality among US whites; however, significantly higher mortality was noted for cancer of the nasopharynx, salivary glands, kidney, esophagus, and cervix (8). Subsequent cancer surveillance efforts showed that overall incidence was below expected rates for 1969–1973 (based on Connecticut cancer incidence rates) but that incidence exceeded expected rates for cancer of the nasopharynx, liver, salivary gland, gallbladder, kidney, and thyroid. Significantly lower incidence rates among AN people compared with US whites were reported for melanoma, lymphoma, and leukemia (9). Since then, we reported emerging patterns in cancer incidence among AN people, such as declines in cervical cancer incidence and increases in lung, breast, and colorectal cancer (CRC) (10–13). Cancer incidence data emphasize the unique patterns of cancer among the AN population compared with other US populations and guide culturally relevant programs in cancer education, prevention, and control.

The term “Alaska Native” refers to 3 broadly defined groups of indigenous people living in Alaska: Eskimo, Indian, and Aleut. AN people comprise approximately 18% of the Alaskan population; 33% live in one of 2 major cities. Health care for AN people was the responsibility of the Indian Health Service (IHS) until 1997, when the Alaska Tribal Health Compact, a self-governance agreement, allowed tribal governments and their regional health corporations to assume this responsibility (14). The objective of this study was to examine cancer incidence and mortality data for AN people from 1970 through 2011. 

## Methods

US data are from the Surveillance, Epidemiology, and End Results (SEER) Program SEER*Stat database and from the SEER Alaska Native Tumor Registry, a population-based registry that includes AN people who lived in Alaska at the time of cancer diagnosis from 1969 to 2011 and who met eligibility requirements for IHS benefits (15). Tumor data and demographic information for AN patients were gathered from electronic medical records, medical charts, pathology reports, and provider dictations from the Alaska Native Medical Center and regional medical facilities. Cancers described in this report include only invasive cancers. Classification of site of cancer origin, histologic cell type, behavior, and grade coding followed the *International Classification of Diseases for Oncology*, second and third editions. Cancer sites of origin were grouped according to SEER site groups. Less than 1% of the cases were identified from death certificates only; 89% of cancers were histologically confirmed. Abstracting followed detailed coding and staging guidelines established by the SEER program, and data were processed through a standard set of computerized edits. SEER data quality is monitored through periodic record review and other SEER data quality-improvement activities. AN population estimates were obtained from the US Census (1970, 1980, and 1990) and from National Center for Health Statistics’ bridged population series for ANs (1981–2010), obtained from the SEER*Stat website (16). Cancer incidence rates were expressed as average annual rates per 100,000 population for a 5-year period, 2007–2011. Age-specific cancer incidence rates were based on data for 2000–2011. US cancer incidence data for 2000–2011 were obtained from a SEER*Stat data set based on 18 registries (17); US trend data for 1973–2011 were obtained from a SEER*Stat data set based on 13 registries (18). AN and US white mortality rates were calculated for years 2007–2011 using data from the National Center for Health Statistics, available from SEER*Stat (19). Incidence and mortality rates, rate ratios (RRs), and corresponding 95% confidence intervals were computed using SEER*Stat statistical software version 8.1.2 (20). Rates were age-adjusted to the US Census 2000 standard population using the direct method for comparison with US white incidence rates. Trend analysis of 2-year averaged incidence rates for 1970–2011 was performed using the Joinpoint Regression Program, version 4.0.4 (21). The Joinpoint regression models determined the best fit for a single line or multisegmented lines across 2-year averaged incidence rates among ANs and US whites. Annual percentage change (APC) in trends was noted for cancers showing significant changes in trend; probability level was set at *P* = .05.

We calculated 11-year average annual age-adjusted death rates for 2 periods: 1990–2000 and 2001–2011. Age-adjustments were performed using the direct method and the 2000 US Census data. Rates were compared and considered significantly different when there was no overlap of confidence intervals.

## Results

Among Alaska men and women, 1,968 cases of invasive cancer were diagnosed during 2007–2011. The age-adjusted incidence rate among AN women for all cancer sites combined was 16% higher than the rate among US white women (RR = 1.16; *P* < .001); however, the rate among AN men was not significantly different from the rate among US white men (RR = 0.94; *P* = .11) (Table 1). The leading cancers among AN and US white populations were lung, colorectal, and prostate cancer among men, and breast, colorectal, and lung cancer among women. We found striking differences in incidence rates between ANs and US whites for several sites. Incidence rates for lung and CRC among ANs exceeded rates among US whites (lung: RR_men_ = 1.59, *P* < .001; RR_women_ = 1.44, *P* < .001; colorectal: RR_men_ = 1.92, *P* < .001; RR_women_ = 2.03, *P* < .001). The prostate cancer rate among AN men was significantly lower than the rate among US white men (RR = 0.48, *P* < .001). Whereas breast cancer rates among AN women were once lower than rates among US white women, we found similar rates (RR = 1.02, *P* = .66). Other disparities between ANs and US whites were observed. Significantly higher rates among ANs were found for the following sites: nasopharynx (RR_men_ = 10.71, *P* < .001; RR_women_ = 10.39, *P* < .001), stomach (RR_men_ = 2.82, *P* < .001; RR_women_ = 3.94, *P* < .001), and kidney (RR_men_= 1.39, *P* = .04; RR_women_ = 1.90, *P* < .001). Significantly lower rates among ANs were found for melanoma of the skin (RR_men_ = 0.09, *P* < .001; RR_women_ = 0.14, *P* < .001 ), urinary bladder (RR_men_= 0.57, *P* = .002), thyroid (RR_women_ = 0.68, *P* = .03), non-Hodgkin lymphoma (RR_men_ = 0.52_,_ *P* = .001), and leukemia (RR_men_ = 0.43, *P* = .002; RR_women_ = 0.53, *P* = .01).

**Table 1:** Average Annual Age-Adjusted Cancer Incidence Rates^a^ per 100,000, Alaska Native and US White Men and Women, 2007–2011

Cancer Site^b^	Men				Women			
	No.	Alaska Native	US White	Rate Ratio (*P* Value)	No.	Alaska Native	US White	Rate Ratio (*P* Value)
**All sites**	899	501.3	532.1	0.94 (.11)	1,069	495.5	424.4	1.16 (<.001)
**Oral cavity and pharynx**	39	18.5	17.0	1.08 (.69)	18	7.8	6.4	1.22 (.47)
Nasopharynx	13	6.5	0.6	10.71 (<.001)	6	2.7	0.3	10.39 (<.001)
**Digestive system**	310	167.2	99.9	1.67 (<.001)	265	129.7	66.6	1.94 (<.001)
Esophagus	9	4.1	8.0	0.50 (.06)	10	4.4	1.7	2.53 (.02)
Stomach	47	26.0	9.2	2.82 (<.001)	38	17.6	4.5	3.94 (<.001)
Colon and rectum	173	95.6	49.6	1.92 (<.001)	153	75.9	37.3	2.03 (<.001)
Colon only	109	64.8	34.3	1.89 (<.001)	115	58.1	27.5	2.11 (<.001)
Rectum only	45	21.1	11.5	1.83 (<.001)	30	14.1	7.1	1.99 (.001)
Liver	28	13.8	10.0	1.37 (.18)	12	5.8	2.9	1.97 (.06)
Gallbladder	3	2.0	0.8	2.66 (.25)	6	3.6	1.4	2.57 (.08)
Pancreas	37	19.1	14.0	1.36 (.10)	28	14.1	10.7	1.31 (.21)
**Respiratory system**	197	123.4	79.6	1.55 (<.001)	152	79.0	55.7	1.41 (<.001)
Larynx	12	7.0	6.0	1.15 (.74)	3	1.2	1.3	0.85 (>.99)
Lung and bronchus	183	115.3	72.4	1.59 (<.001)	148	77.6	53.8	1.44 (<.001)
**Bones and joints**	3	0.9	1.2	0.75 (1.0)	1	0.3	0.8	0.36 (.65)
**Soft tissue and heart**	5	2.8	4.2	0.66 (.47)	11	4.2	2.8	1.48(.30)
**Skin, excluding basal and squamous**	8	3.4	35.3	0.09 (<.001)	9	4.3	21.4	0.20 (<.001)
Melanoma of the skin	7	3.1	32.3	0.09 (<.001)	6	2.8	20.0	0.14 (<.001)
**Breast**	1	0.7	1.2	0.54 (.72)	303	131.6	128.0	1.02 (.66)
**Female genital system**	—	—	—	—	113	47.5	50.5	0.94 (.56)
Cervix uteri	—	—	—	—	30	11.2	7.8	1.42 (.08)
Corpus uteri	—	—	—	—	41	16.6	25.4	0.65 (.006)
Ovary	—	—	—	—	28	13.4	13.0	1.03 (.92)
**Male genital system**	140	76.6	147.7	0.51 (<.001)	—	—	—	—
Prostate	119	67.5	139.9	0.48 (<.001)	—	—	—	—
Testis	18	7.2	6.6	1.08 (.79)	—	—	—	—
**Urinary system**	89	52.8	62.5	0.84 (.15)	53	26.2	21.0	1.24 (.16)
Urinary bladder	34	22.5	39.4	0.57 (.002)	9	5.2	9.5	0.54 (.07)
Kidney and renal pelvis	55	30.3	21.7	1.39 (.04)	44	21.0	11.0	1.90 (<.001)
**Eye and orbit**	3	2.2	1.1	2.07 (0.42)	0	—	—	—
**Brain and nervous system**	11	4.7	8.4	0.55 (.08)	19	7.0	6.0	1.17 (.56)
**Endocrine system**	12	5.4	7.7	0.70 (.31)	38	15.2	21.1	0.72 (.05)
Thyroid	11	5.2	6.9	0.75 (.45)	35	14.0	20.4	0.68 (.03)
**Lymphoma**	30	13.8	28.2	0.48 (<.001)	31	14.2	19.8	0.71 (.08)
Non-Hodgkin lymphoma	27	13.1	24.9	0.52 (.001)	30	13.8	17.2	0.80 (.27)
**Myeloma**	8	5.2	7.2	0.71 (.43)	5	2.2	4.3	0.50 (.15)
**Leukemia**	18	7.7	17.5	0.43 (.002)	17	5.8	10.7	0.53 (.01)
**Mesothelioma**	1	0.3	2.1	0.16 (.004)	0	—	—	—
**Kaposi sarcoma**	2	1.3	0.9	1.51 (.81)	1	0.6	0.1	7.54 (.25)
**Undefined**	22	14.5	10.5	1.37 (.25)	33	19.8	7.9	2.51 (<.001)

a Source of data: Surveillance Epidemiology and End Results (SEER) program SEER*Stat database (17).

b Not all cancer site subgroups are shown for the major cancer site groups.

### Age-specific rates

Age-specific incidence rates for all cancer sites combined were similar among AN and US white men for most 10-year age groups (Table 2). AN women aged 50 to 79 had significantly higher rates than US white women of similar age, and AN men aged 50 to 69 had significantly lower rates than US white men of similar age. Stomach and CRC incidence rates, which are 2 or more times higher among ANs than among US whites, showed disparities across nearly all 10-year age groups. Disparities in lung cancer rates were greatest among the older age groups. Breast cancer rates among AN women were similar to rates among US white women for half of the 10-year age groups. Prostate cancer rates were much lower among AN men aged 50 to 79 than among US white men of similar age. Kidney cancer incidence among ANs by age group was generally similar to rates among US whites, but rates were higher among AN men aged 70 to 79 than US men of similar age and among AN women aged 50 to 59 than US white women of similar age.

**Table 2:** Age-Specific Cancer Incidence Rates^a^ per 100,000 Population, Alaska Native and US White Men and Women, 2000–2011

Cancer Site/Age Group, y	Men				Women			
	No.	Alaska Native Rate	US White Rate	Rate Ratio (*P* Value)	No.	Alaska Native Rate	US White Rate	Rate Ratio (*P* value)
**All sites**								
30–39	84	98.4	81.6	1.20 (.11)	135	159.2	147.2	1.08 (.39)
40–49	195	214.6	211.8	1.01 (.88)	358	392.4	355.7	1.10 (.07)
50–59	424	646.4	712.6	0.90 (.04)	550	818.4	684.0	1.19 (<.001)
60–69	535	1,556.8	1,841.0	0.84 (<.001)	552	1,519.3	1,234.4	1.23 (<.001)
70–79	485	2,827.2	2,938.4	0.96 (.41)	470	2,272.4	1,788.4	1.27 (<.001)
≥80	188	3,217.2	3,212.3	1.00 (>.99)	209	2,210.8	1,978.5	1.11 (.12)
**Stomach**								
30–39	5	5.8	1.4	4.08 (.02)	11	13.2	1.2	11.13 (<.001)
40–49	20	21.8	4.1	5.30 (<.001)	11	11.9	2.7	4.44 (<.001)
50–59	23	35.1	11.2	3.14 (<.001)	11	16.4	5.3	3.07 (<.001)
60–69	22	67.0	28.1	2.38 (<.001)	20	55.0	11.2	4.89 (<.001)
70–79	25	151.0	51.3	2.94 (<.001)	18	87.9	22.6	3.89 (<.001)
≥80	12	222.4	72.2	3.08 (<.001)	4	42.0	36.4	1.15 (.91)
**Colon and rectum**								
30–39	13	15.2	6.7	2.25 (.01)	8	9.3	6.0	1.55 (.30)
40–49	35	38.5	22.9	1.67 (.01)	52	57.0	19.7	2.89 (<.001)
50–59	78	118.9	71.6	1.66 (<.001)	79	117.5	51.5	2.28 (<.001)
60–69	106	303.5	160.4	1.89 (<.001)	87	241.0	106.7	2.25 (<.001)
70–79	93	540.4	293.9	1.83 (<.001)	109	528.3	209.2	2.52 (<.001)
≥80	35	611.1	402.4	1.51 (.03)	47	497.7	323.2	1.54 (.01)
**Lung**								
30–39	3	3.5	2.2	1.60 (.58)	1	1.1	2.5	0.43 (.70)
40–49	20	21.7	16.8	1.29 (.31)	20	21.5	17.0	1.26 (.35)
50–59	83	126.4	78.1	1.61 (<.001)	53	78.9	63.6	1.23 (.14)
60–69	114	328.8	262.2	1.25 (.02)	102	283.4	197.3	1.43 (<.001)
70–79	143	827.3	505.8	1.63 (<.001)	119	577.1	344.5	1.67 (<.001)
≥80	46	785.8	524.9	1.49 (.01)	42	446.7	300.0	1.48 (.02)
**Breast**								
30–39	—	—	—	—	48	57.2	43.8	1.30 (.09)
40–49	—	—	—	—	166	182.5	154.4	1.18 (.04)
50–59	—	—	—	—	203	302.1	260.7	1.15 (.04)
60–69	—	—	—	—	162	444.7	398.1	1.11 (.18)
70–79	—	—	—	—	87	420.0	460.4	0.91 (.43)
≥80	—	—	—	—	23	244.4	412.3	0.59 (.01)
**Prostate**								
30–39	2	2.4	0.5	5.03 (.12)	—	—	—	—
40–49	11	11.8	22.4	0.52 (.03)	—	—	—	—
50–59	65	98.9	210.8	0.46 (<.001)	—	—	—	—
60–69	101	292.5	681.2	0.42 (<.001)	—	—	—	—
70–79	63	366.5	899.7	0.40 (<.001)	—	—	—	—
≥80	28	468.9	642.7	0.72 (.11)	—	—	—	—
**Kidney**								
30–39	6	7.3	3.8	1.92 (.19)	4	4.7	2.6	1.78 (.38)
40–49	13	14.3	13.6	1.05 (.93)	5	5.5	7.4	0.74 (.68)
50–59	29	44.2	33.8	1.30 (.19)	24	35.7	16.6	2.14 (<.001)
60–69	30	87.5	68.4	1.27 (.22)	15	40.3	32.5	1.24 (.47)
70–79	29	170.3	93.8	1.81 (<.001)	14	65.8	46.2	1.42 (.25)
≥80	7	108.9	91.2	1.19 (.75)	9	96.3	44.3	2.17 (.05)

a Source of data: Surveillance Epidemiology and End Results (SEER) program SEER*Stat database (17).

### Cancer time trends

Joinpoint analysis indicated a 1.1% annual increase in combined cancer incidence rates among AN men and women during 1970–2005 (Table 3). The annual rate of increase among AN women (1.2%) was greater than the rate among AN men (0.7%) during 1970–2011. All site rates among US white men increased 1.5% annually during 1973–1989 and decreased 0.5% annually during 1995–2011. Rates among US white women increased during 1973–1982 (0.5% annually) and 1985–1999 (0.5% annually); no significant declines were found.

**Table 3:** Joinpoint Analyses for Trends Among Alaska Native (1970–2011) and US White (1973–2011) 2-Year Averaged Annual Cancer Incidence Rates — Model Selections

Site/Sex	Trend 1	APC^a^^, ^ b	Trend 2	APC^a^^, ^ b	Trend 3	APC^a^^, ^ b	Trend 4	APC^a^^, ^ b
**All Sites**								
**Men and women**								
Alaska Native	1970–2005	1.1^b^	2005–2011	−1.9	—	—	—	—
US white	1973–1982	0.9^b^	1982–1991	1.7^b^	1991–2008	−0.2^b^	2008–2011	1.7
**Men**								
Alaska Native	1970–2011	0.7^b^	—	—	—	—	—	—
US white	1973–1989	1.5^b^	1989–1992	4.7	1992–1995	−3.7	1995–2011	−0.5^b^
**Women**								
Alaska Native	1970–2011	1.2^b^	—	—	—	—	—	—
US white	1973–1982	0.5^b^	1982–1985	2.6	1985–1999	0.5^b^	1999–2011	−0.2
**Lung**								
**Men and women**								
Alaska Native	1970–1985	7.2^b^	1985–2011	0.2	—	—	—	—
US white	1973–1981	2.9^b^	1981–1990	1.3^b^	1990–2007	−1.6^b^	2007–2011	−2.6^b^
**Men**								
Alaska Native	1970–1995	2.8^b^	1995–2011	−1.5	—	—	—	—
US white	1973–1978	2.6^b^	1978–1988	0.3	1988–2007	−1.6^b^	2007–2011	−2.9^b^
**Women**								
Alaska Native	1970–1985	12.9^b^	1985–2011	0.9	—	—	—	—
US white	1973–1982	6.3^b^	1982–1991	3.5^b^	1991–2007	0.6^b^	2007–2011	−2.5^b^
**Colorectal**								
**Men and women**								
Alaska Native	1970–1999	1.6^b^	1999–2011	−2.6^b^	—	—	—	—
US white	1973–1985	0.9^b^	1985–1995	−2.0^b^	1995–1998	2.1	1998–2011	−2.9^b^
**Men**								
Alaska Native	1970–1997	2.0^b^	1997–2011	−1.9	—	—	—	—
US white	1973–1986	1.1^b^	1986–1995	−2.3^b^	1995–1998	1.8	1998–2011	−3.3^b^
**Women**								
Alaska Native	1970–2005	0.9	2005–2011	−7.3	—	—	—	—
US white	1973–1984	0.7^b^	1984–1995	−2.0^b^	1995–1998	2.4	1998–2011	−2.6^b^
**Breast**								
**Women**								
Alaska Native	1970–1997	5.1^b^	1997–2011	−0.3	—	—	—	—
US white	1973–1980	−0.5	1980–1987	3.8^b^	1987–1999	0.5	1999–2011	−0.8^b^

a Alaska Native annual percentage change (APC) based on 2-year averaged incidence rates.

b APC significantly different from zero at α = .05.

**Figure 1:**
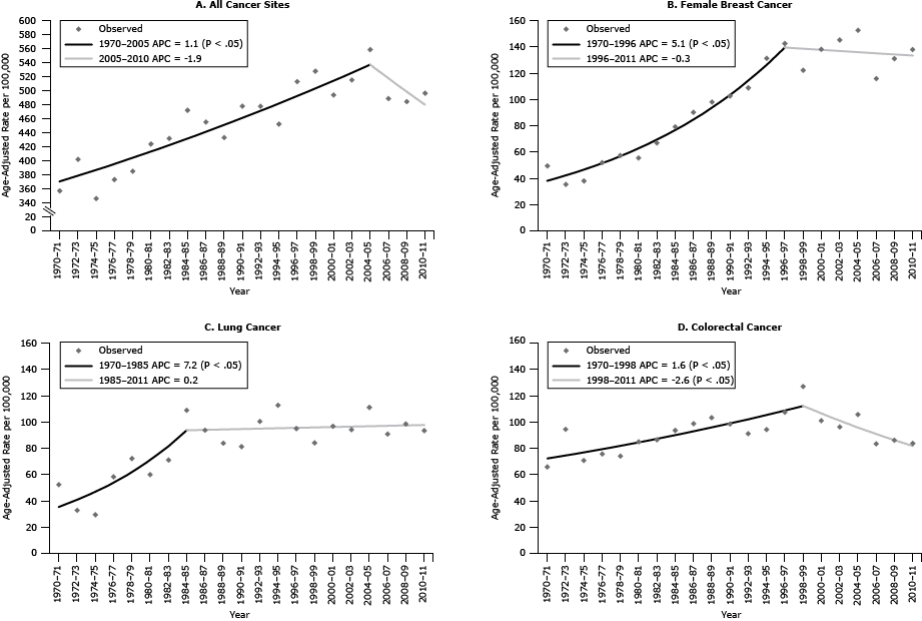
Joinpoint regression analysis to detect changes in incidence trends among Alaska Native people, 1970–2011. A, All cancer sites combined, men and women. B, Breast cancer, women. C, Lung cancer, men and women. D, Colorectal cancer, men and women. A *P* value <.05 indicates a significant annual percentage change (APC) in trend. Source: Alaska Native Tumor Registry.

Joinpoint analysis of incidence rates by cancer site groups indicated significant increases in rates among men and women of both races. Only lung cancer rates among US whites showed any significant declines. Significant increases of 1.6% annually in CRC incidence among AN men and women combined were found for 1970–1999. Significant annual declines of 2.6% in CRC incidence among AN men and women combined were found for 1999–2011 (Figure 1). Declines in CRC among AN men or women were not significant. Declines of 2% annually in rates of CRC among US white men and women were found for 1985–1995, 10 or more years earlier than the decline among ANs.

Breast cancer rates among AN women increased significantly by 5.1% annually during 1970–1997. No significant increases or decreases in breast cancer incidence rates among AN women were found for later years. Rates among US white women increased 3.8% annually during 1980–1987 and later declined by 0.8% annually during 1999–2011.

### Cancer mortality rates

**Figure 2:**
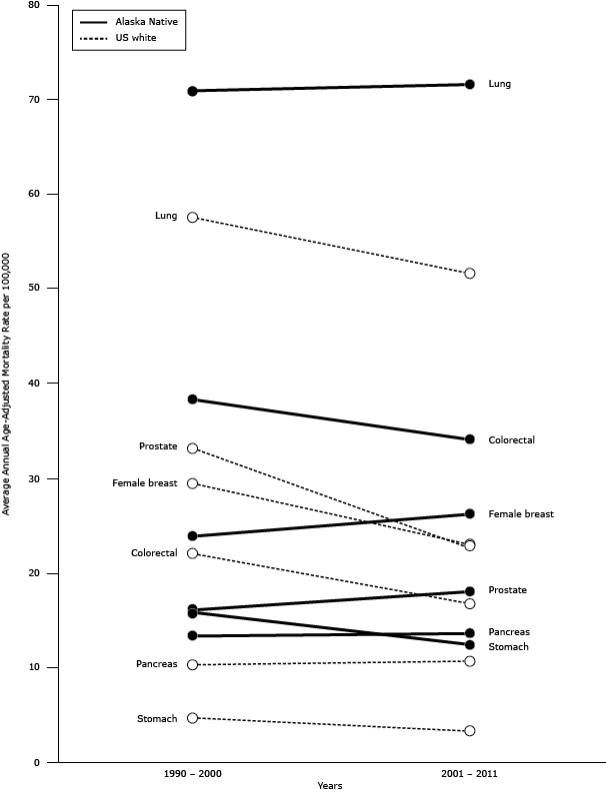
Cancer mortality rates in Alaska Native and US white populations for 2 periods, 1990–2000 and 2001–2011. **Table:** 

No change was found in the cancer mortality rate for all sites combined among AN people between the 2 periods examined (1990–2000 and 2001–2011). Mortality rates among AN people did not change over time for the leading cancers (lung, colorectal, and breast cancer) (Figure 2). However, mortality rates among US whites declined significantly by the second period for all cancer sites combined (lung, colorectal, breast, and prostate cancer). Lung cancer was the leading cause of cancer death for both periods and both races. Lung and CRC mortality rates among ANs were significantly higher than rates among US whites for both periods.

Cancer Site	Race	1990–2000	2001–2011
**Lung**	Alaska Native	71.0	71.6
	US white	57.5	51.7
**Colorectal**	Alaska Native	38.3	34.1
	US white	22.0	16.8
**Female breast**	Alaska Native	23.9	26.2
	US white	29.4	23.0
**Prostate**	Alaska Native	16.1	18.0
	US white	33.1	22.7
**Pancreas**	Alaska Native	13.3	13.6
	US white	10.3	10.6
**Stomach**	Alaska Native	15.8	12.5
	US white	4.7	3.3

## Discussion

High cancer incidence rates among AN people compared with rates among US whites were first reported in the early 1970s. During the past 40 years, lung cancer and breast cancer incidence among AN people increased by 200%, and CRC incidence increased by 35%. Significant declines in cancer incidence and mortality in the United States began in 1992 among men and in 1998 among women, primarily in the 4 leading cancers: lung, colorectal, prostate, and breast cancer (22). Rates of leading cancers among ANs did not decline, except for CRC incidence and mortality rates; this situation has resulted in an ever-increasing disparity in cancer incidence and mortality among the AN population.

Patterns of cancer incidence and mortality are not similar among American Indian and AN (AI/AN) groups in the United States. Incidence rates for all cancer sites combined among AI/AN groups are lower than rates among US whites; however, AI/AN cancer rates by IHS region show striking differences between AI/AN groups and US whites. Underreporting of cancer incidence because of race misclassification was demonstrated in several studies that linked cancer registry and IHS registration data (23–26). Studies on cancer conducted among AI/AN living in US counties containing tribal lands (or areas adjacent to tribal lands), where contract health services are provided for AI/ANs and where rates of race misclassification are lower, showed higher rates of certain cancers among AI/ANs in some IHS regions compared with rates among non-Hispanic whites from the same counties (27–29). The contract health services delivery area (CHSDA) counties grouped by IHS region showed higher incidence rates of lung cancer in the regions of the Northern Plains and Alaska; higher incidence rates of CRC in the regions of the Northern Plains, Southern Plains and Alaska; higher incidence rates of kidney cancer in the regions of the Northern Plains, Southern Plains, Southwest, and Alaska; and higher incidence rates of stomach cancer in the regions of the Northern Plains, Southern Plains, Pacific Coast, Southwest, and Alaska among AI/ANs than among non-Hispanic whites from the same regions (30–32). These studies underscore the disparity between AI/ANs and US whites and demonstrate geographic differences in cancer incidence.

Lung cancer rates among ANs have not declined as they have among US whites (22). High rates of lung cancer are likely to continue until tobacco use declines significantly. Since 2001, the Alaska Behavioral Risk Factor Surveillance System (BRFSS) survey has consistently measured rates of 38% to 45% for current smokers among the AN population — nearly double the rate among Alaska whites (33). Youth Risk Behavior Surveillance (YRBS) surveys in 2007, 2009, and 2011 among high school students in Alaska indicated that one-fourth to one-third of AN students were current smokers. Far fewer non-Native Alaska students in Alaska reported they were current smokers (10%–13%) in the same surveys (34).

In 2003, Alaska Native Tribal Health Consortium (ANTHC) established a Tobacco Prevention and Control Program to build capacity for tribal health organizations of the Alaska Tribal Health System to develop, expand, or revitalize nicotine-dependence treatment services. Later, tobacco treatment specialists who received educational training on tobacco cessation became community-based resources for providing information on quitting tobacco and counseling to those who had decided to quit. Training of these specialists continues.

The Healthy People 2020 target for the proportion of nonsmokers in the United States is 88% (35), and the Healthy Alaskans 2020 target for the proportion of Alaskans who are tobacco-free is 83% for adults (from the current baseline of 78%) (36). A 10% increase in the number of tobacco-free ANs would result in a tobacco-free rate of approximately 68%, a rate more achievable than the state goal during the next 6 years.

CRC incidence and mortality among US men declined rapidly after 1998 and among women after the 1970s (37). We found a significant decrease of 2.6% annually in CRC incidence rates among AN men and women combined for 1999–2011. The recent decrease in incidence of CRC is probably due to a recent increase in colorectal screening efforts among AN people. Beginning in the early 1990s, when high incidence rates of CRC were documented, the ANTHC began endoscopy training for nurse practitioners and physician assistants, an effort to make endoscopy more available (38). In 2007, ANTHC supported itinerant screening colonoscopy field clinics. ANTHC also began an outreach program to follow up and encourage colonoscopy for eligible members of families of patients diagnosed with CRC. This program added a patient navigator to encourage people due for colonoscopy to make a screening appointment. In addition, the patient navigators assisted people by scheduling precolonoscopy appointments, assisting with travel plans, sending out appointment reminders, and answering questions about the bowel preparation and procedure (39). As a result, the CRC screening rate among AN people 50 years or older increased from 29% in 2000 to 65% in 2011 (12). A grant awarded by the Centers for Disease Control and Prevention to individual Alaska tribal health organizations provides patient navigators in medical centers at several regional hubs to assist AN people in rural areas in accessing CRC screening. Because CRC screening has increased only during recent years, significant reductions in CRC mortality are not expected immediately.

National data indicate that breast cancer mortality has not declined among AI/AN women in the United States (40). Breast cancer incidence and mortality declines in US white women are attributed to decreases in the use of hormone replacement therapy, increases in screening by mammography, early detection of in situ disease, and improvements in breast cancer treatment. Breast cancer incidence rates were once low among AN women (13,41) but then increased dramatically from 1970 to 1997; they have since remained constant. Rates of mammography (mammography in the past 2 years) among AN women aged 50 to 74 who had a mammogram in the past 2 years (76.8%) are similar to rates among white women in Alaska (74.9%), according to BRFSS surveys for 2004–2012 (33). Thirty percent of AN women with breast cancer are diagnosed when they are younger than 50. Age-specific breast cancer incidence rates among AN women younger than 50 were 14% to 23% higher than rates among US white women in our analysis, although significantly different rates were found only among women aged 40 to 59. In light of these data, changing screening recommendations for mammography to an earlier age may be worthy of consideration. Further study is needed to understand how risk factors such as race, gravidity and parity, age at first mammogram, screening history, stage at diagnosis, use of hormone replacement therapy, and family history of breast cancer have influenced breast cancer incidence and mortality among AN women.

The US Healthy People 2020 target is to reduce death rates by 10% for all cancer deaths from 179 to 161 deaths per 100,000 people. The Healthy Alaskans 2020 target to reduce cancer death rates is 162 deaths per 100,000, an 8% reduction over the baseline rate of 176 per 100,000 (36). The AN cancer mortality rate for 2001–2011 was 246 per 100,000 for all cancer sites combined, 36% higher than the rate among US whites (181 per 100,000). A recent review indicated that cancer mortality among ANs has not changed, whereas rates among US whites declined in the past 2 decades (22). It is unlikely that AN people will meet the state or national 2020 targets of cancer mortality reduction, especially because smoking rates have not decreased.

CRC and breast cancer screening programs appear to be having an impact on controlling breast cancer incidence and reducing CRC incidence rates. It is not known how many people would benefit from increased screening efforts, but BRFSS data from survey years 2004–2012 combined indicated that approximately 23% of AN women aged 50 to 74 did not receive a mammogram in the 2 years before the telephone interview. BRFSS survey data from combined years 2010–2012 also indicated that 40% of AN men and women aged 50 or older have not had a sigmoidoscopy or colonoscopy.

The impact of the CRC screening program may contribute to future declines in CRC mortality rates. Improvements in treatment of cancer may also contribute to the relatively stable rates seen for breast and lung cancer. The impact of tobacco cessation programs is not detectable on tobacco use trends among AN adults according to BRFSS data. Further study is needed to examine changes in smoking behavior in regions where smoking cessation programs are most active.

Our study has several limitations. Classification of cancer cases by AI/AN race was largely determined by eligibility for tribal health services. Denominator data were obtained from the US Census; participants self-identified as a single race in census years 1970, 1980, and 1990. Beginning with the Census 2000, individuals could choose more than one race, and the combination of AI/AN plus another race inflated the expected population counts for AI/AN in Alaska by about 10%. Several adjustments were made to correct for the overestimate of the AN population, including the process of “bridging,” which allowed a comparison of data before and after the Census 2000. Although eligibility for tribal health services can be identified for cases, it cannot be determined from census data.

Many of our incidence and mortality counts had small values, and differences between rates were difficult to detect. Combining several years of data is often needed to provide reliable estimates, but shifts in trends may not be identified for several years when using this approach. Because of the small number of deaths, we could not perform Joinpoint analysis on AN mortality rates.

The objective of this study was to describe patterns in cancer incidence and mortality and trends over time among AN people, by age and sex, and to compare these data with US data to identify disparities. Our data provide information to assess the impact of cancer control interventions (such as colonoscopy in reducing CRC). Cancer incidence rates paired with risk factor data may provide communities with information from which cancer prevention programs may be built. We hope to provide a resource for health care providers, tribal leaders, communities, and individuals who work to improve the health of AN people.

Disparities in cancer persist among AN people. Overall cancer incidence rates for both sexes combined among AN people exceed rates among the US white population, largely because of a 16% excess rate among AN women. Rates among AN men and women are higher than rates among US whites for multiple site-specific cancers: lung, stomach, colorectal, kidney, and nasopharynx. Rates among ANs increased for all cancers and selected sites; whereas rates among US whites decreased — increasing cancer disparities. Further studies are needed to identify the causes for the unique cancer pattern and ways to enhance cancer control.
